# Genomics and Proteomics Analyses Revealed Novel Candidate Pesticidal Proteins in a Lepidopteran-Toxic *Bacillus thuringiensis* Strain

**DOI:** 10.3390/toxins12110673

**Published:** 2020-10-26

**Authors:** Ayda Khorramnejad, Joaquín Gomis-Cebolla, Reza Talaei-Hassanlouei, Yolanda Bel, Baltasar Escriche

**Affiliations:** 1 Departamento de Genética/ERI BioTecMed, Universitat de València, Burjassot, 46100 València, Spain; ayda.khoramnejad@uv.es (A.K.); joaquin.gomis-cebolla@uantwerpen.be (J.G.-C.); 2 Department of Plant Protection, College of Agriculture and Natural Resources, University of Tehran, Karaj 31578-77871, Alborz, Iran; rtalaei@ut.ac.ir; 3 Ecosystem Management Research Group, Department of Biology, University of Antwerp, BE-2610 Wilrijk, Belgium

**Keywords:** LC-MS/MS, *Plodia interpunctella*, *Grapholita molesta*, genome sequencing, insect bioassay, characterization, Mpp proteins, Xpp proteins

## Abstract

Discovery and identification of novel insecticidal proteins in *Bacillus thuringiensis* (Bt) strains are of crucial importance for efficient biological control of pests and better management of insect resistance. In this study, the Bt strain KhF, toxic for *Plodia interpunctella* and *Grapholita molesta* larvae, underwent genomics and proteomics analyses to achieve a better understanding of the bases of its pathogenicity. The whole-genome sequencing results revealed that the KhF strain contained nine coding sequences with homologies to Bt insecticidal genes. The lepidopteran toxic mixture of spores and crystals of this Bt strain was subjected to liquid chromatography and tandem mass spectrometry (LC-MS/MS) to assess the protein composition. The results of the proteomic analyses, combined with the toxin gene sequences, revealed that two of the main components of the crystals were two new candidate pesticidal proteins, named KhFA and KhFB. These proteins showed a similarity lower than 36% to the other known Bt toxins. The phylogenetic analysis showed that the KhFA and KhFB grouped with the newly denominated Xpp and Mpp (former ETX/Mtx) pesticidal protein groups, respectively. Altogether, this study has led to the discovery of two novel candidate pesticidal toxins in the lepidopteran toxic KhF strain.

## 1. Introduction

*Bacillus thuringiensis* (Bt) is the most successful and widely used microbial control agent for the management of insect pests [1]. This entomopathogenic bacterium produces parasporal inclusion bodies comprised of crystal (Cry) and cytolytic (Cyt) proteins upon the sporulation [2,3]. Due to the importance of Bt in the management of insect pests, many studies have focused on the development of Bt-based bio-insecticides as well as transferring Bt genes into the agricultural crops [4,5]. Indeed, several Bt strains have been characterized so far based on their genome sequencing and/or protein composition following mass-spectrometry based approaches to identify their gene content and proteome, as well to predict their biological activity [6,7,8,9,10,11,12]. Molecular based characterization methods, through proteomics- and/or genomics-based analyses, have provided a rapid and comprehensive strategy for the identification of Bt toxins [13,14]. On the other hand, the extensive application of Bt products and Bt crops has led to the emergence of resistance to Bt toxins in insects [15,16]. Therefore, the identification of novel Bt strains and toxins may enhance the potency of Bt-based bioinsecticides and the efficiency of Bt crops. Thus, the discovery of new Bt genes or proteins with different biological activity, a wider spectrum of activity, and higher insecticidal potency can lead to better management of insect pests.

The whole genome of newly isolated Bt strains is being sequenced to speed up the detection of new putative insecticidal Bt genes [9,10]. Indeed, genome sequencing has been employed by many researchers to describe pesticidal and virulence factors genes [11,12,17,18,19] as a fast and efficient means of Bt strain characterization.

On the other hand, it is believed that the insecticidal activity of Bt strains is mostly attributed to the type, amount, and relative abundance of the proteins produced [20,21]. Hence, proteomics analysis accelerates the screening studies and can reveal the protein composition of the Bt parasporal crystals, provided that the detected protein sequences exist in the protein databases. Different proteomic approaches enable the researchers to investigate the protein profile of Bt strains for different purposes such as the phylogenetic classification of *Bacillus* spp. [22], Bt protease profile [23], bacteriocin characterization [24], spore proteomes [25], crystal protein composition [26,27], the protein composition of solubilized spore/crystal mixture [6], proteome involved in carbon metabolism [28], the metabolic regulation of sporulation and crystal formation [29,30], and the protein composition of whole Bt cell [31]. Also, several studies have coupled whole-genome sequencing with the liquid chromatography and tandem mass spectrometry (LC-MS/MS) to investigate the gene content, the insecticidal proteins, and the bioactive compounds of newly isolated or highly potent Bt strains [8,18,32].

The Bt strain KhF was originally isolated from a soil sample in Iran. We had observed that the spore/crystal mixture of KhF was toxic for second instar larvae of *Plodia interpunctella* (Lep.: Pyralidae). Following the conventional solubilization processes, the crystal proteins of the KhF strain could not be completely solubilized. The PCR-based method was employed to screen the presence of Bt toxin genes belonging to the main family groups (*cry1*, *cry1Aa*, *cry1Ab*, *cry1Ac*, *cry1Ad*, *cry1Ag*, *cry1C*, *cry1D*, *cry1H*, *cry1I*, *cry1G*, *cry2*, *cyt1*, *cyt2*, *vip1*, *vip2*, *vip3*, *ps1*, *ps2*, *ps3*, *ps4*, *sip1,* and *sip2* genes). The results revealed only the possible presence of the *cry2* gene [21]. These characteristics alongside the insecticidal activity of the KhF spore/crystal mixture against lepidopteran *P. interpunctella* led us to conclude that most probably KhF harbored new Bt genes and proteins different from the known ones. Therefore, in this study, genomics and proteomics approaches were coupled and used to identify the possible toxic agents in the spores and crystals mixture. Due to the applicability of LC-MS/MS analysis incorporated with full genome sequencing in the identification of new Bt proteins, our results allowed us to identify two novel Bt proteins.

## 2. Results

### 2.1. Crystal Morphology, Protein Profile, and Insecticidal Activity of KhF Strain

**Figure 1:**
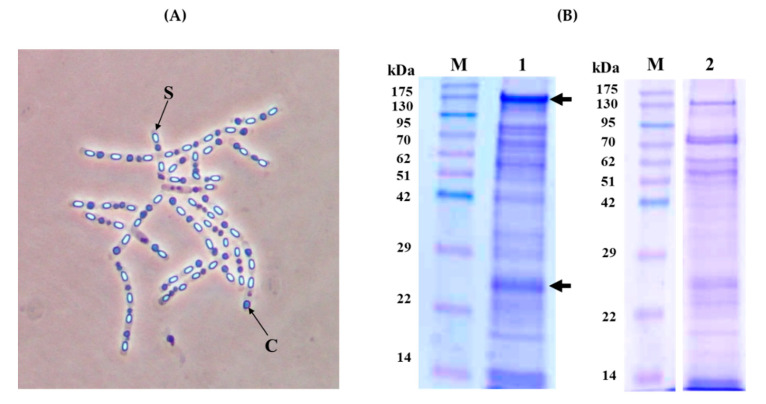
Crystal morphology and SDS-PAGE analysis of the KhF strain. (**Panel A**) Phase-contrast microscopy (×100 magnification). Letters “S” and “C” point to spore and crystal, respectively. (**Panel B**) SDS-PAGE analysis. Lane 1; Spore/crystal mixture, lane 2; partially solubilized KhF crystals in 50 mM carbonate buffer (pH 10.5). Lanes M; molecular mass marker (Pink pre-stained protein ladder, Nippon genetics). The arrows point to the major bands of 130 and 23 kDa.

*B. thuringiensis* strain KhF was originally isolated from a soil sample in Iran [21]. Phase-contrast microscopy of the bacterial cells, sampled during the sporulation phase before sporangium lysis, showed that the KhF strain produced spherical crystals (Figure 1, Panel A).

After completion of sporulation, the protein profile of the spore/crystal mixture of KhF strain was analyzed by sodium dodecyl sulfate-polyacrylamide gel electrophoresis (SDS-PAGE) analysis, showing two main bands of almost 130 and 23 kDa and several other minor bands (Figure 1, Panel B, Lane 1). The solubilization of the crystals, performed in the usual conditions for Bt crystal (Cry) proteins (solubilization buffer 50 mM Na_2_CO_3_, 0.1 M NaCl, and 10 mM dithiothreitol, pH 10.5), allowed a partial solubilisation of the sample, with the SDS-PAGE profile shown in Figure 1, Panel B, Lane 2. As a result, the bands of 130 kDa and 23 kDa were present, with others of a similar intensity ranging from about 70 to 50 kDa. To achieve a complete solubilization of KhF crystals, different buffers with pHs ranging from 4 to 12 were used, as well as different temperatures or incubation times (data not shown). None of the employed conditions improved the solubilization process of the crystal components.

The insecticidal activity of the KhF strain was assessed by first feeding instar larvae of *Ostrinia nubilalis*, *Grapholita molesta*, *Spodoptera exigua,* and *Helicoverpa armigera* with KhF spores/crystal mixtures at two different concentrations (10^8^ and 10^9^ spore/mL). Based on the bioassay results, *G. molesta* larvae were found to be susceptible to the spore/crystal mixture of KhF (Table 1). Meanwhile, neither considerable toxicity nor growth inhibition was observed in *O. nubilalis*, *S. exigua,* and *H. armigera* larvae after treatment with the spore/crystal mixture of KhF strain at the tested concentrations (Table 1).

### 2.2. Genome Sequencing, Gene Annotation, and Phylogenetic Analysis

The genomics analysis of the KhF strain allowed the identification of putative pesticidal Bt toxin genes and the detection of the virulence factors that might be present in this strain. The whole-genome sequencing (with the accession number of SRR12105151) resulted in a total 1.814.544 high-quality reads, with an average length of 150 bp. These reads were assembled in 135 scaffolds. The assembled paired reads resulted in a genome size of 5.9 Mb, with an N50 of 120.35 kb (length of the shortest contig at 50% of the total genome length) and a GC content of 35%. The longest scaffold length was 302.89 kb. The coding sequence prediction of the assembled reads showed that the 135 scaffolds defined a total of 6272 putative coding sequences (CDS) that represented 99.19% of the length of the bacterial genome and contained 82.76% the annotated genes and 16.43% of the hypothetical genes. In addition, the genome of KhF contained 62 tRNAs (0.001% of the bacterial genome length) and 123 repeated regions (0.007% of the bacterial genome length) (Table 2). In addition, the completeness of the assembly was evaluated with Busco [33] using the bacterial lineage data set of conserved single-copy orthologs. The Busco results showed 99.3% of genome completeness and 2.0% and 0.7% of duplicated and missing genes, respectively.

**Figure 2:**
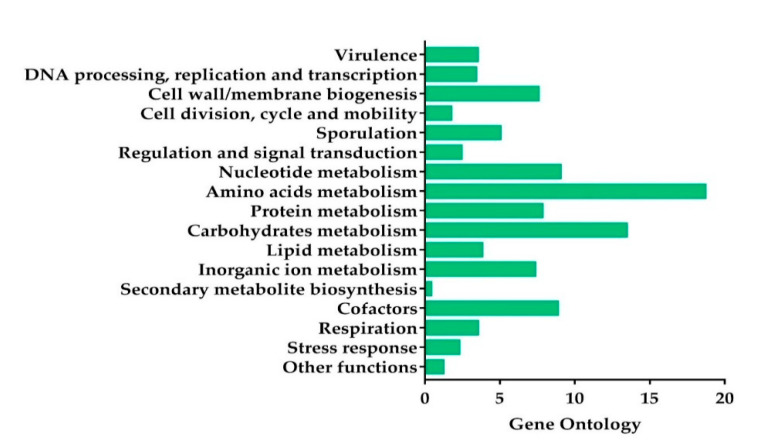
Subsystem category distribution of the KhF strain genes by the genome annotation based on the Rast server. The *x*-axis represents the percentage of the predicted encoding genes associated with a subsystem.

Based on the genome annotation results, 2236 predicted CDS were grouped in the 17 main categories (Figure 2). The most abundant genes were the ones associated with amino acid and carbohydrate transport and metabolism, followed by nucleotide metabolism, cofactors, protein metabolism, cell/wall membrane biogenesis, inorganic ions metabolism, and sporulation, in decreasing order (Figure 2).

**Figure 3:**
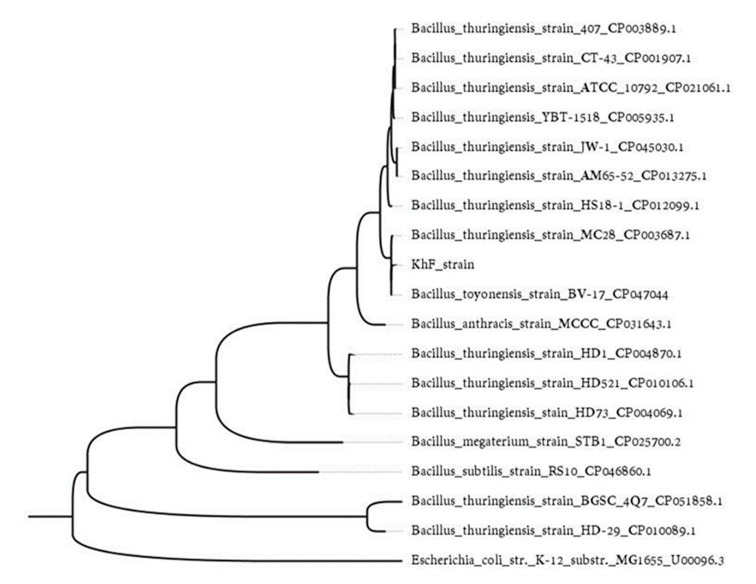
Maximum likelihood tree displaying similarity in the *gyrB* gene sequence of the KhF strain and other *Bacillus* strains. Branch lengths represent the number of substitutions per site of the multiple-sequence alignment as a measure of divergence. The tree was rooted based on *Escherichia coli* as an outlier.

To determine the taxonomic relation of KhF with other *Bacillus* spp. (*B. thuringiensis*, *Bacillus cereus, Bacillus megaterium,* and *Bacillus anthracis*), we used the sequence of the gyrase subunit B (*gyrB*) gene. As a result, the KhF strain clustered with other *B. thuringiensis* strains within a subgroup with *B. thuringiensis* MC28 and *Bacillus toyonensis* strain BV-17 (Figure 3).

### 2.3. Search for Virulence Factors and Bacterial Toxins in the Genome

The putative insecticidal protein genes in the KhF strain were detected using the Btoxinscanner [13] and Blastx tools. For the Blastx analysis, two different databases were used: (1) the Basic Local Alignment Search Tool (NCBI) and (2) a customized Bt protein database [8] generated from the Bt toxin list available in the Bt nomenclature website [34]. The results are summarized in Table 3.

The analyses of the whole KhF genome using Btoxinscanner retrieved two genes (*peg5520* and *peg5936*, Table 3). The Blast analysis using NCBI only retrieved the gene sequence *peg5936*, which encoded a protein homologous to a known Bt bacterial toxin, the WP_153593625.1 from the ETX/Mtx2 group (Table 3). Nevertheless, by using the customized Bt protein database generated from the Bt toxins list [8], it was possible to find seven coding sequences (*peg5520*, *peg5936*, *peg309*, *peg2096*, *peg3627*, *peg5521*, and *peg5522*) that encoded proteins with homology to the Vip family proteins, as well as another coding sequence (*peg4213*)*,* of which its product shared a 24% homology with the Cry49Aa protein (Table 3). All the proteins encoded by these genes showed high homologies (from 80% to 100%), with “hypothetical proteins” described in the NCBI databases but not defined as bacterial toxins (Table 3). The multiple sequence alignment analysis of the candidate insecticidal protein genes indicated that the *peg2096* and *peg309* genes showed a sequence similarity of 96% and their closet homolog is Vip1Aa1.

The *peg5937* gene was detected and included in the table after gene synteny evaluation of the retrieved toxin coding genes. The *peg5937* was located upstream of the *peg5936*, a putative *vip1A* gene that usually is accompanied by a *vip2* gene in the same scaffold (Figure 3). The protein encoded by *peg5937* showed no homology to any of the known Bt toxins available in the customized Bt toxins database (Table 3 and Table S1). However, it showed more than 89% homology with the hypothetical protein WP_153593624.1 from Bt (Table 3).

**Figure 4:**
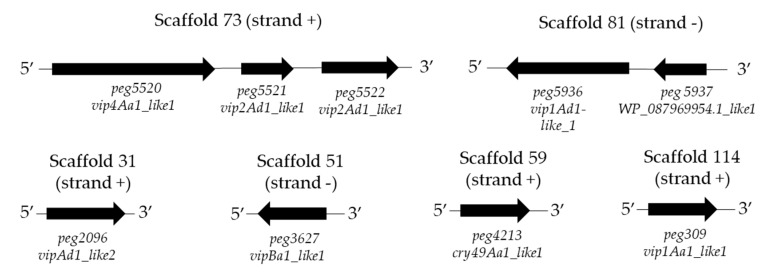
Gene synteny of the candidate insecticidal protein genes detected in the genome of the KhF strain. Strand (+) indicates the respective genes in the positive DNA strand. Strand (−) shows the respective genes in the negative DNA strand.

The results of the candidate insecticidal genes synteny showed that the nine retrieved coding sequences were located in six different scaffolds. The *peg5520, peg5521,* and *peg5521* genes were grouped in scaffold 73 and the *peg5936* and *peg5937* genes were localized in scaffold 81. In addition, the presence of *rho*-independent terminators (ARNfold) was determined in scaffold 73 and 81 (Figure 4), suggesting that at least the *peg5520* contains an erpin terminator sequence at contig position 3554 (5′ AATAATTAGACgcgatttcaaatagagttgatattaggaatcgcGTTCTTTTTTTCAA 3′). For the rest of the genes, no sequences similar to erpin terminator were detected.

Aside from the Bt pesticidal protein genes, the KhF strain harboured 35 coding sequences belonging to different classes of virulence factors (Table S1). A number of 10 coding sequences with similarity to other toxins present in *Bacillus* spp., such as haemolytic enterotoxin (HE) and non-haemolytic enterotoxin (NHE), were also detected (Table S1).

### 2.4. Proteomics of Spores and Crystals

The LC-MS/MS analysis of the spore/crystal mixture proteins was carried out to investigate the proteins expressed by the KhF strain that provoked toxicity to lepidopterans. The complete output of the LC-MS/MS was analysed against a general protein database (SwissProt) and against a specific customized protein database generated from the KhF coding sequences predicted by the genome sequencing analysis.

**Figure 5:**
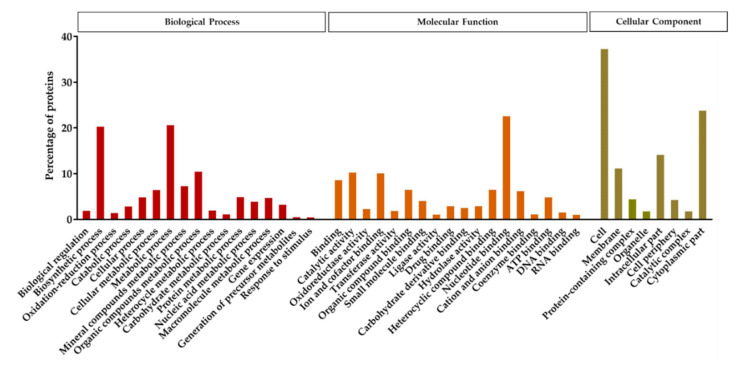
Functional annotation and classification of the proteins detected in the spore/crystal mixture of KhF strain, after LC-MS/MS analysis. The function of the identified proteins was classified into three main groups, representing the biological process, the cellular component, and the molecular function.

Protein analysis using the custom specific protein database resulted in the identification of 434 proteins (6.72% of the genome coding sequences), 327 of which showed a confidence value higher than the 99% (Unused Score > 2). Out of the 327 proteins, 83.5% protein sequences were annotated to the GO term. The detected proteins were classified into three different functional groups; biological process, molecular function, and cellular component (Figure 5). The functional annotation indicated that in the biological process groups, proteins related to the biosynthetic and metabolic processes were the most numerous. Among the proteins categorized in the group of molecular functions, the nucleotide binding proteins were the most abundant. Based on the cellular components, the majority of the detected proteins grouped in the cell, cytoplasmic part, intracellular part, and membrane subgroups.

To gain more insight into the protein composition of the KhF spore/crystal mixture, the results of LC-MS/MS analysis were annotated with SwissProt database. The functional composition of the annotated proteins showed several classifications, namely, pathogenesis, sporulation proteins, protein biosynthesis, metabolism, and other functions (Table 4). In the pathogenesis group, Immune Inhibitor A (InhA) and bacillolysin were found, which affect the insect innate immune responses and consequently, the Bt virulence [31]. The Spo0A (stage 0 sporulation protein A) and SpoVG, regulatory proteins in the initiation of *Bacillus* spp. sporulation, were sorted in the sporulation proteins group. The elongation factors involved in the protein biosynthesis were also identified.

The LC-MS/MS analysis showed that 17.45% of the total detected peptides belonged to a set of 7 proteins (Table 5), while 82.54% of the peptides represented the rest (427) of the proteins (the protein identification is supported by less than 1% of the total peptides). Therefore, from a semi-quantitative point of view (based on the number of peptides detected), the results show that few proteins constitute the most abundant proteins and the rest should be considered as a trace.

The expression of the predicted insecticidal genes found in the genome of the KhF strain was investigated, comparing genomics and proteomics data. As a result, two predicted candidate insecticidal proteins, encoded by *peg5936* and *peg5937* genes (Acc. No. MT701514 and MT701521, respectively), were found based on the Protein Pilot v5.0. The protein identification was supported by the presence of at least one specific peptide (Table 4 and Table S2). The Peg5936 protein was estimated as the most abundant, representing 79.15% of the total protein content, while the Peg5937 protein accounted for 4.05% of the total protein (Table 6).

The *peg5936* gene encodes a 1223 amino acid protein with 67.7% similarity to the ETX/Mtx2 family Bt toxin and *peg5937* gene encodes a 216 amino acid protein, showing 89.4% similarity to a hypothetical protein from Bt (Table 3). The Peg5936 protein (from now on called KhFB) showed the highest match (identity 28%, E-value 1.9^−37^) to the “binary_toxB domain-containing protein” from UniProt database with the accession number A0A1V0DG95. Amongst the Bt toxin proteins, the Peg5937 protein (from now on called KhFA) matched to the “uncharacterized 20 kDa protein in CryB1 5′ region of *B. thuringiensis* subsp. *kurstaki*” from UniProt database with the accession number P21732 (identity 30%, E-value 3.5^−32^).

The three-dimensional structure of Peg5936 and Peg5937 proteins was investigated by using Phyre2 [37]. An appropriate model for Peg5936 and Peg5937 proteins could not be generated due to the low sequence identity of the given templates (less than 30% identity) and low similarity of these proteins to the known Bt proteins. However, the partial structural analysis of these proteins showed that 24.6% and 14% of Peg5937 protein are comprised of α-helices and β-strands, respectively, and Peg5936 protein has 17.8% and 19.2% of α-helices and β-strands, respectively, in its structure. Not all the regions of amino acid sequences of Peg5936 and Peg5937 proteins could be modelled due to their novelty.

### 2.5. Cluster Tree Analysis of the KhFA and KhFB Toxins

**Figure 6:**
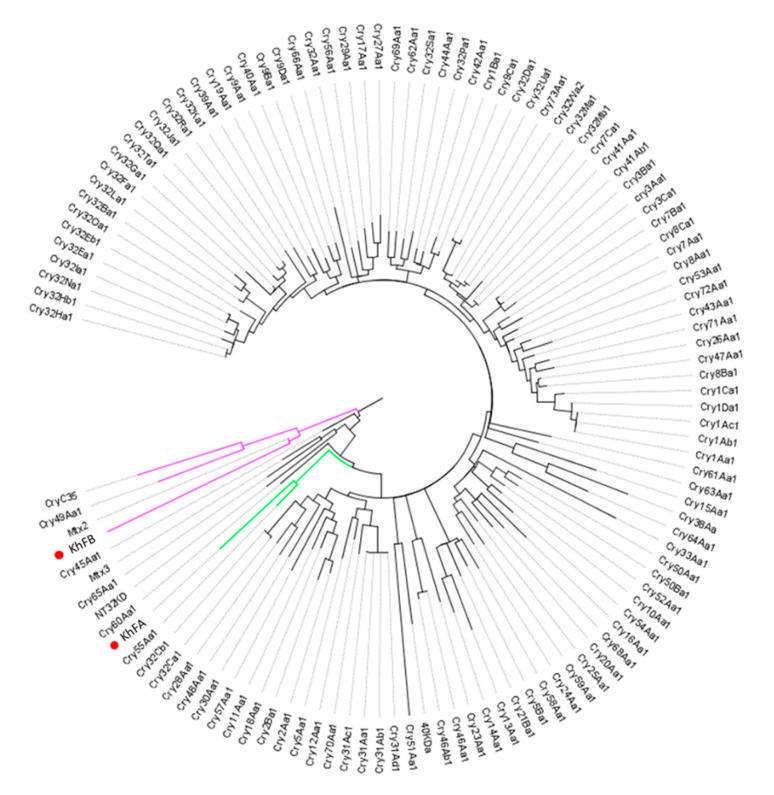
Dendrogram showing the relationship of KhFA (Peg5937) and KhFB (Peg5936) with other *Bacillus* pesticidal proteins. The red dots indicate the position of the new proteins in the dendrogram. Branch lengths represent the number of substitutions per site of the multiple-sequence alignment as a measure of divergence.

The analysis of KhFB (Peg5936) protein showed that this protein clusters with Mtx2, recently classified in the Mpp group of bacterial pesticidal proteins [38]. The KhFA (Peg5937) protein falls into a cluster with Cry55Aa1, a Bt structurally uncharacterized pesticidal protein that belongs to the newly defined Xpp group [38] (Figure 6). As the similarity of KhF proteins to the respective closest homologs is lower than 45%, the Peg5936 and Peg5337 proteins could be considered new Bt-like proteins.

## 3. Discussion

Bt based insecticides are eco-friendly alternatives for pest control. The emergence of insect resistance to Bt toxins is one of the major constraints that threaten the application of Bt in the microbial control of pests. Therefore, the identification of novel Bt strains or toxins is of scientific and economic interest and is critical for efficient control of insect pests and the management of insect resistance.

The KhF was originally isolated from a soil sample in Iran. The strain produces spherical crystals and the spores/crystals mixture was found to be toxic for *P. interpunctella* second instar larvae. Our previous studies have shown that this strain did not produce β-exotoxin (a Bt toxin analogous to ATP, which has a general toxic effect on all living organisms) and the PCR-screening showed that the strain probably carried *cry2* genes but not *cry1*, *cry1Aa*, *cry1Ab*, *cry1Ac*, *cry1Ad*, *cry1Ag*, *cry1C*, *cry1D*, *cry1H*, *cry1I*, *cry1G*, *cry2*, *cyt1*, *cyt2*, *vip1*, *vip2*, *vip3*, *ps1*, *ps2*, *ps3*, *ps4*, *sip1,* and *sip2* genes [21]. Another characteristic of this strain is that only partial solubilization of crystals could be achieved. Due to the observation of the insecticidal activity against *P. interpunctella* and incomplete solubility of KhF crystals, the discovery of novel Bt toxins in this strain was very promising. Therefore, the gene content and the protein composition of the spore/crystal mixture of this Bt strain have been investigated in this work.

The entire genome of KhF strain was sequenced through the Illumina NextSeq500 Sequencer. The genome assembly results and a subsequent search using two different databases revealed that the KhF strain comprises nine candidate insecticidal Bt proteins with the closest homology to Bt hypothetical protein, ricin-domain, and ETX/Mtx2 proteins based on the non-redundant protein database of NCBI and to Vip1Aa1, Vip1Ad1, Vip1Ba1, Vip2Ac1, Vip2Ad1, Vip4Aa1, and Cry49Aa1 toxins (with low similarity values ranging from 24% to 36%) based on the customized Bt toxins database (Table 3). It is worth mentioning that following PCR-based screening of KhF strain gene content, the amplification of *vip1* and *vip2* genes was not observed [21]. Therefore, the low similarity of *peg5936*, *peg309*, *peg2096*, *peg3627*, *peg5521*, and *peg5522* genes to their corresponding *vip1* or *vip2* genes reinforce the novelty of the detected genes.

The candidate insecticidal protein genes detected in the genome of KhF were localized in six different scaffolds. Alongside the described candidate insecticidal Bt genes, the KhF strain contains 35 CDS encoding different classes of pathogenic factors (Table S1). Among them, 10 coding sequences showed similarity to other known toxins present in *Bacillus* spp., such as haemolytic enterotoxin (HE) and non-haemolytic enterotoxin (NHE). Due to the close phylogeny of *B. cereus sensu lato* group, the haemolysin and non-haemolysin enterotoxin genes are conserved amongst the *Bacillus* spp. and indeed, the presence of these genes has been reported in different Bt strains [19,39]. The genomic analysis of Bt strain HD-1 revealed the presence of the haemolysin Xh1A family genes and it has been proposed that these genes might play an important role in the toxicity process of HD-1 strain [19].

The phylogenetic study of the KhF strain constructed based on the *gyrB* gene showed that this Bt strain might have a close relationship with *B. thuringiensis* strain MC28 and *B. toyonensis* BV-17 (Figure 3). According to the whole-genome sequencing, the *B. thuringiensis* strain MC28 has three plasmids and harbours different *cry* and *cyt* genes, namely *cry4Cc1*, *cry30Fa1*, *cry53Ab1*, *cry54Aa1*, *cry54Ab1*, *cry68Aa1*, *cry69Aa1*, *cry69Aa2*, *cry70Ba1*, *cyt1Da1*, and *cyt2Aa3* [40]. The other closely related *Bacillus* strain to KhF is *B. toyonensis* BV-17, as a non-pathogenic probiotic *Bacillus* species [41,42]. The haemolysin BL secreted as three components by the *B. toyonensis* BV-17 showed cytotoxicity against cancer cells [42]. Interestingly, *peg3633*, *peg3634*, and *peg3632* genes from the KhF strain showed 100% similarity to haemolysin BL lytic component L1, component L2, and binding component, respectively.

The toxicity of the KhF strain has been assessed through bioassays, using mixtures of spores and crystals that are toxic for *P. interpunctella* [21] and *G. molesta* (Table 1). The protein composition of the spore/crystal mixture of the KhF strain was determined by LC-MS/MS analysis. It is worth mentioning that the Vip proteins are not present in crystals since they are produced and secreted to the media before the sporulation phase differently to the proteins that are present in the parasporal crystals. Under this point of view, seven out of the nine candidate insecticidal protein genes detected after genomic analysis in the KhF strain would not be detected in crystals.

In the proteomics analyses, a total of 434 proteins were identified in the spore/crystal mixture of KhF. After eliminating the redundant proteins, 327 specific proteins with a confidence level of higher than 99% were detected. These proteins were compared to the translated proteins deduced from the KhF sequenced genome. To ensure the certainty of the existence of a detected protein in the strain, the proteins with the several discriminating peptides showing a confidence value higher than 99% that did not match with other detected proteins with a higher score were selected. According to such principles, among the insecticidal Bt genes detected in the genomics analysis, only two of them, *peg5936* and *peg5937*, were expressed and identified in the spore/crystal mixture of the KhF strain and constituted a high percentage of the total protein content in the crystals (Table 6). Moreover, two bands of 130 and 23 kDa coinciding with the predicted size of these two new genes, named KhFB (*peg5936*) and KhFA (*peg5937*), were visualized in the SDS-PAGE analysis of KhF crystals (Figure 1, Panel B).

KhFA and KhFB proteins can be considered as new Bt-like proteins, different to the known Cry, Cyt, Vip, or Sip proteins because of their low similarity with the known Bt proteins. The phylogenetic analysis indicated that KhF proteins grouped with Mtx2 and Cry55Aa belong, respectively, to the ETX/Mtx2-like (now called Mpp) and the structurally unclassified Bt pesticidal proteins (now called Xpp) groups. None of the proteins of these groups are three domain Cry proteins, the most well-known pesticidal Bt toxins [2].

Mtx2 family toxin, produced by some *Lysinibacillus sphaericus* and Bt strains, exhibits mosquitocidal activity against different dipteran species [43]. ETX/Mtx2 proteins have a β-sheet structure and are considered as pore forming pesticidal toxins [44]. The toxic activity of Cry55 against nematodes and coleopterans has been reported [45,46], while neither the structural homolog nor the mode of action of this protein is known [2,44]. Due to the homology of Peg5937 to Cry55 protein and the existence of metalloproteinase as a toxic factor for nematodes [47], the KhF strain may be considered as a nematicidal strain. Further experiments are required to investigate the insecticidal/nematicidal activity of the KhF strain.

Besides the Bt toxins, several virulence factor genes have been detected in the KhF strain. It has been demonstrated that in addition to the pesticidal toxins, Bt strains are capable of producing different pathogenic factors influencing the mechanism of action of a Bt strain [19,48]. The Immune Inhibitor A (InhA), bacillolysin, Spo0A and SpoVG proteins, enolase, and cold-shock proteins were found in the spore/crystal mixture of the KhF strain following the LC-MS/MS analysis. It has been demonstrated that the InhA and bacillolysin are metalloproteases produced during the expression of *cry* genes at the early in sporulation phase in a Bt strain [7,31,49,50] and they may affect the insect immune responses [51,52,53,54]. Moreover, the proteomics results revealed the presence of enolase in the KhF strain and the possible function of this enzyme in the virulence of Bt strains has also been speculated [25]. Besides the virulence factors, the Spo0A, SpoVG, and cold-shock proteins produced during the sporulation phase [7] and involved in the sporulation process of *Bacillus* spp. [30,55,56,57] were detected.

The bioassays revealed the toxicity of the spore/crystal mixture of KhF strain for *P. interpunctella* and *G. molesta.* The susceptibility of *P. interpunctella* to different Bt proteins (Cry1A, Cry1B, Cry1C, Cry1D, Cry1E, Cry1F, Cry1I, Cry2A, Cry9, Cry39, and Cry40) has been demonstrated [58,59]. As well, the toxicity of Cry1A, Cry1Ca, Vip3Aa, and Vip3Af Bt toxins to *G. molesta* has been shown [60]. According to the PCR-based screening [21] and the whole-genome sequencing and proteomics results presented in this work, these genes and their subsequent proteins are not present in the KhF strain. Nevertheless, the genomics study of the strain together with the proteomics analyses of spores and crystals have highlighted the presence of KhFA and KhFB, two novel putative Xpp and Mpp-like proteins, described for the first time in this study. These proteins, most probably together with some bioactive virulence factors found in the KhF strain, could cause the insecticidal activity of KhF spores and crystals mixtures. Further experiments will be required to assess the individual insecticidal potential of each one of these two new proteins.

## 4. Conclusions

In this study, the molecular description of a Bt strain (KhF) at both levels of gene content and protein composition of the spore/crystal mixture has been presented. The whole genome sequencing was integrated with the LC-MS/MS analysis. Based on the genome sequencing results, the KhF strain harbours eight coding sequences, showing homology to Bt *cry* and *vip* toxins. The expression of the detected insecticidal Bt genes was confirmed by LC-MS/MS analysis. The results showed that two new predicted pesticidal proteins, named Peg5936 (KhFB) and Peg5937 (KhFA), were found abundantly in the crystals of the KhF strain. These new proteins showed a similarity lower than 36% to other known Bt toxins. According to the cluster tree analysis, the new proteins grouped with the Mpp and Xpp-like proteins. The proteomic analysis also rendered 327 different proteins including Bt pesticidal toxins and virulence factors, which may influence the pathogenicity of KhF strain against different pests. Altogether, the proteomic/genomic approach employed in this study has led to the discovery of two novel candidate Bt toxins. Further experiments are required to assess the pesticidal activity of these two new Bt proteins.

## 5. Materials and Methods

### 5.1. Microscopic Observation, Protein Profiling, and Spore/Crystal Mixture Preparation

The wild-type *B. thuringiensis* strain KhF was isolated from a soil sample based on the acetate selective method [61] in Iran [21]. Phase-contrast microscopy of sporulated cultures of the KhF strain was used to determine crystal morphology. To produce spore/crystal mixtures for bioassays, a single colony of KhF strain was grown in CCY medium [62] for 48 h at 29 °C with 180 rpm. Bacterial cells were sampled at 48 h of incubation and observed microscopically to confirm the presence of parasporal inclusions. The culture was heated at 70 °C for 20 min and used for inoculation of the final culture, which was incubated at 29 °C till completion of sporulation and sporangium lysis. The released spores and crystals were collected following Estela et al. [63]. Briefly, spores and crystals were harvested by centrifugation at 6000× *g* for 10 min at 4 °C. The pellet was washed twice with 1 M NaCl and 10 mM EDTA and with 10 mM KCl, and was centrifuged at 24,000× *g* for 10 min at 4 °C. For bioassays, the spore/crystal mixture were resuspended in distilled Milli-Q water. For solubilization of crystal proteins, the spore/crystal pellet was suspended in solubilization buffer (50 mM Na_2_CO_3_, 0.1 M NaCl, and 10 mM dithiothreitol, pH 10.5) and incubated for 2 h at room temperature with gentle shaking. Protein profiles were observed by sodium dodecyl sulfate 12% polyacrylamide gel electrophoresis (SDS-PAGE) [64]. For spores and crystals quantification, a Neubauer chamber haemocytometer (Brand GMBH, Germany) was used.

To increase protein solubilization of crystals of the KhF strain, buffers ranging from pH of 3.6 to 12 were used (50 mM potassium acetate buffer (pH 3.6–5.6), 20 mM Tris-HCl buffer (pH 7–9), and 50 mM carbonate buffer (pH 10–12)). Two different reducing agents, 10 mM dithiothreitol (DTT) and/or 0.2% β-mercaptoethanol, were also used to cleave disulfide bonds. Solubilizations were performed at room temperature (RT) or 37 °C for different time intervals of 5 min, 2, 16, and 24 h (data not shown).

### 5.2. Insect Colonies

Four different lepidopteran insect species, *Ostrinia nubilalis* (Crambidae), *Grapholita molesta* (Tortricidae), *Spodoptera exigua* (Noctuidae), and *Helicoverpa armigera* (Noctuidae), were employed in this study. The insect colonies were reared on the artificial diets [65,66,67,68] and maintained in the insect chamber at 25 ± 1 °C, with a photoperiod of 16:8 (L:D) h and a relative humidity of 70 ± 5%.

### 5.3. Toxicity Assays

The insecticidal activity of spores and crystals suspension of KhF strain was assessed against *O. nubilalis*, *G. molesta*, *S. exigua*, and *H. armigera* first instar larvae. The bioassay experiments were conducted based on the surface contamination method at two different concentrations of 10^8^ and 10^9^ spore/mL of spore and crystal mixture. Control larvae were treated with distilled Milli-Q water. The toxicity assays were repeated at least three times using 16 larvae in each replicate. The mortality and functional mortality (number of dead larvae plus the number of the first instar larvae) were recorded after 7 days.

### 5.4. DNA Isolation, Genome Assembly, and Annotation Analysis

Total DNA was extracted using the DNeasy Blood & Tissue Kit (Qiagen, Hilden, Germany) following manufacturer’s instructions. The purified DNA was quantified by Nanodrop 2000 (Thermo Scientific, Waltham, MA, USA) and the homogeneity of the DNA was evaluated by 1% agarose gel electrophoresis.

Genome sequencing was performed with the Illumina NextSeq500 Sequencer by the Genomics Research Hub facility (Cardiff University, Wales, UK). The Genomics Research Hub facility trimmed and eliminated the low quality reads (limit of 0.01) and deletion of ambiguous nucleotides (maximal 2 nucleotides allowed). Afterwards, the reads were assembled using the CLC genomic workbench 10.1.1 (Qiagen^®^ Company, Aarhus, Denmark) by the *de novo* assembly tool and default parameters with a minimum contig length of 200 bp. The completeness of the assembly was evaluated by Busco v3 [33] using the bacterial odb9 lineage dataset. In addition, the assembled reads were annotated with Rast server [69] and the coding sequence (CDS) prediction was performed with the Glimmer v2 [69].

To determine the putative insecticidal protein genes, three different approaches were used. In the first approach, the Btoxinscanner tool was used to predict the putative insecticidal protein genes [13]. In the second approach, the predicted coding sequences were firstly filtered against a customized Bt protein database [8] made based on the Bt toxin list available in the Bt nomenclature website [34] and Blastx was performed using the criteria of genetic code bacteria and archaea, e-value 0.01, and word size 6 [70]. In the third approach, a Blastx from NCBI was performed, applying a series of filters to the output of the first Blasts search: (1) from the output, the coding sequences with a hit length >100 aa and bit score >50 were selected, and (2) the selected coding sequences were compared to the non-redundant database. The virulence factors of the KhF strain were predicted using the VFanalyzer tool [71] (File S1). In addition, the presence of signal peptide were predicted by SignalIP server 4.0 [72] for all the candidate insecticidal protein genes and virulence factors.

### 5.5. Protein Identification by LC-MS/MS Analysis

The detection of the proteins in the spore/crystal mixture of the KhF strain was done by LC-MS/MS at the proteomics facility of the SCSIE at the University of Valencia. The process of sample preparation and digestion of spore/crystal mixture proteins were performed as described elsewhere [73], with slight modifications. Briefly, the sample was digested with sequencing grade trypsin (Promega, Madison, WI, USA) as mentioned in the following steps. The sample was solubilized in 50 mM ammonium bicarbonate in a final volume of 400 µL containing 2.5 mM DTT and incubated for 20 min at 60 °C. The thiol groups were alkylated by 5.5 mM of iodoacetamide (IAM) in 50 mM ammonium bicarbonate for 30 min at room temperature in the dark. The excess of IAM was quenched with 11 mM DTT at 37 °C for 30 min. The samples were digested overnight with 500 ng of trypsin (ratio 1:10; w:w) at 37 °C. The digestion was stopped with 6 µL of 10% trifluoroacetic acid (TFA) in water. The sample was concentrated by speed vac to reach the final volume of 12 µL. The protein identification of the spore/crystal mixture was carried by the Paragon algorithm [74] via the Protein Pilot v 5.0 (ABSciex, Madrid, Spain) against two different databases—UniProt database and also a customized database that has been created with all the coding sequences predicted by Glimmer v2 software for the KhF strain higher than 100 aa. Protein Pilot v 5.0 default parameters (trypsin specificity, cys-alkylation, taxonomy restricted to human, and the search effort set to through) were used to search in the homemade protein database.

In addition, the functional annotation of the identified proteins was performed with the SwissProt Database using the Blast2GO v5.0 software (BioBam bioinformatics, Valencia, Spain) [75].

### 5.6. Phylogenetic Analyses

The phylogenetic analyses were done with the MEGA V6 software (Molecular Evolutionary Genetics Analysis, State College, PA, USA) [76] for both the DNA gyrase subunit B (*gyrB*) gene and the KhF toxins (Peg5936 and Peg5937). The sequence *gyrB* gene is being used in the phylogenetic classification studies as a sufficient discriminatory single gene in *B. cereus* group, *B. thuringiensis* strains, and serovars [77]. The nucleotide sequences of the *gyrB* gene available in the microbial database (https://www.ncbi.nlm.nih.gov/genome/browse/#!/prokaryotes/486/) were aligned with Clustal W and trimmed with trimAi [78]. The *Bacillus* pesticidal protein sequences were aligned by using MUSCLE [79] and trimmed with trimAI. The default parameters were used for both alignments and trimming. The curated alignments were used to generate a maximum likelihood tree (default software parameters) and bootstrapped using 1000 replicates. Finally, the phylogenetic trees were exported to Newick format and edited with Figtree [80].

### 5.7. Availability of Data and Material 

The raw data of the KhF strain have been deposited at Sequence Read Archive (SRA): SRR12105151. The DNA and protein sequences of the detected candidate insecticidal genes in the KhF strain are deposited in the GenBank with the following accession number; *peg5520* (MT701513), *peg5936* (MT701514), *peg2096* (MT701515), *peg309* (MT701516), *peg3627* (MT701517), *peg4213* (MT701518), *peg5521* (MT701519), *peg5522* (MT701520), *peg5937* (MT701521).

## Figures and Tables

**Table 1:** Insecticidal activity of spore/crystal mixture of KhF against different lepidopteran species.

Insect Species	Spores/mL	% Mortality ^1^	% Functional Mortality ^1^
*G. molesta*	10^8^	59 ± 13	87 ± 7
	10^9^	90 ± 14	95 ± 7
*O. nubilalis*	10^8^	3 ± 3	3 ± 3
	10^9^	3 ± 3	3 ± 3
*H. armigera*	10^8^	6 ± 6	6 ± 6
	10^9^	6 ± 6	6 ± 6
*S. exigua*	10^8^	0 ± 0	0 ± 0
	10^9^	7 ± 6	19 ± 9
*P. interpunctella* ^2^	10^8^	42 ± 2	-

^1^ Values represent the mean ± standard error, ^2^ Data adapted from Khorramnejad et al., 2018 [21]. - Not analyzed.

**Table 2:** Summary of the automated genome annotation of the KhF strain by the Rast server.

Annotation	Features	Gene Content	% Gene Content ^1^	Length (Mb)	% Length ^2^
Total	Genome content ^3^	6457	-	4.99	(100)
	tRNAs	62	-	0.006	0.001
	Repeat region	123	-	0.035	0.007
Blast search	Total sequences ^4^	6272	(100)	4.95	99.19
	Annotated genes ^5^	4494	71.65	4.13	82.76
	Hypothetical genes ^6^	1778	28.35	0.82	16.43

^1^ The gene content values refer to the total predicted putative coding sequences (annotated genes, hypothetical genes, and repeat region) predicted by the Rast server. ^2^ The length values refer to the total genome content. ^3^ Predicted coding sequences plus tRNAs. ^4^ Predicted coding sequences plus repeated regions. ^5^ Predicted sequences that showed a blast hit in the database of the RastServer. ^6^ Predicted sequences that did not show a blast hit in the database of the RastServer. - Not applicable.

**Table 3:** Candidate insecticidal protein genes detected in the KhF strain.

Gene Identity	Length (aa)	Scaffold	Closest Homolog	Similarity (%)	Coverage (%)	E-Value	SignalIP Server
*peg5520* ^1^	867	73	WP_098163495.1|hypothetical protein ^N^	99.54	100	0.0	Yes
			AEB52299.1|Vip4Aa1 ^C^	35.81	57.83	6.0^−93^	
*peg5936* ^1^	1223	81	WP_153593625.1|ETX/Mtx2 family from Bt ^N^	67.67	100	0.0	No
			AGC08395.1|Vip1Ad1^C^	26.9	60.60	1.0^−21^	
*peg309*	333	114	WP_088083339| hypothetical protein ^N^	97	100	0.0	Yes
			ADK79125.1|Vip1Aa1 ^C^	27.41	59.26	4.0^−12^	
*peg2096*	566	31	WP_000734347.1|hypothetical protein ^N^	100	100	0.0	Yes
			AGC08395.1|Vip1Ad1 ^C^	25.62	58.68	5.0^−11^	
*peg3627*	739	51	WP_000811547| hypothetical protein ^N^	100	100	0.0	Yes
			AAR40886.1|Vip1Ba1 ^C^	27.97	58.05	2.0^−12^	
*peg4213*	347	59	WP_098475832| RICIN domain-containing protein ^N^	80.35	100	0.0	Yes
			CAH56541.1|Cry49Aa1 ^C^	23.98	67.54	7.0^−25^	
*peg5521*	184	73	PHE82359.1| hypothetical protein ^N^	97.81	100	5.0^−124^	No
			CAI40768.1|Vip2Ad1 ^C^	27.88	61.21	9.0^−13^	
*peg5522*	502	73	WP_098163497.1|hypothetical protein ^N^	99	100	0	Yes
			AAO86513.1|Vip2Ac1 ^C^	28.64	60.56	8.0^−14^	
*peg5937*	216	81	WP_153593624.1|hypothetical protein from Bt ^N^	89.39	92	1.0^−127^	No
			- ^2^	-	-	^-^	

^N^ NCBI database; Blastx search results using the Non Redundant protein database of the NCBI. ^C^ Customized database; Blastx search results using a customized protein database performed using the Bt toxin nomenclature database. ^1^ The genes *peg5520* and *peg5936* have been predicted using the tool Btoxinscanner and Blastx. ^2^ The coding sequence *peg5937* was not detected in the blast search against the customized Bt toxins database. - Not applicable.

**Table 4:** Detected proteins in spore/crystal mixture of KhF strain by LC-MS/MS analysis.

Category	Protein Description	Accession Number	Matched Peptides ^1^	Sequence Coverage ^2^
Pathogenesis	Immune inhibitor A	P23382	156	50
	Bacillolysin	P05806	10	16
Sporulation proteins	Putative septation protein	A9VN61	14	57
	Stage 0 sporulation protein A	P52933	5	27
Protein biosynthesis	60 kDa chaperonin	Q4MPR6	20	32
	1-pyrroline-5-carboxylate dehydrogenase	A9VRG6	28	40
	Homocysteine methyltransferase	B7IVP2	20	22
	10 kDa chaperonin	Q81VE2	22	48
	RNA-binding protein	Q65JA8	7	21
	Elongation factor	B7IT17	5	16
	Hut operon positive regulatory protein	Q81Y44	4	38
	Serine hydroxymethyltransferase	A9VSB4	6	15
	Leucine dehydrogenase	P0A393	4	14
	Phosphoribosyl-ATP pyrophosphatase	Q6HLE1	6	13
	Glutamine synthetase	P19064	18	21
	Purine biosynthesis protein	Q6HPA0	14	30
	Translation initiation factor	Q81VQ7	5	76
Metabolism	Methylmalonate semialdehyde dehydrogenase	B7IW48	85	57
	Enolase	B7IP20	70	65
	Glucose-6-phosphate isomerase	Q816G0	16	41
Other functions	DNA protection	Q8RPQ1	45	67
	Aminopeptidase	Q81XS5	37	37
	Superoxide dismutase	Q81LW0	38	90
	Pyridoxal 5′-phosphate synthase	A9VM99	31	55
	Cytosol aminopeptidase	Q816E3	32	37
	Purine nucleoside phosphorylase	Q81T09	8	21
	Major cold shock protein	Q81TW8	2	22

^1^ The number of distinct matching peptides having at least a 95% level of confidence. ^2^ The percentage of matching amino acids to the detected peptides having confidence greater than or equal to 95% divided by the total number of amino acids in the sequence.

**Table 5:** The most abundant proteins in the spore/crystal mixture of the KhF detected by LC-MS/MS analysis.

Gene Identity	Unused ProtScore	Sequence Coverage (%)	Matched Peptides	Function
*peg5936*	188.93	92.96	171	WP_153593625.1|ETX/MTX2 family from Bt
*peg5610*	40.00	74.18	23	MULTISPECIES: elongation factor Tu [Bacillaceae]|WP_001029617.1
*peg6159*	40.09	35.65	22	MULTISPECIES: PrkA family serine protein kinase [Bacillaceae]|WP_000353271.1
*peg3524*	26.07	67.07	20	MULTISPECIES: N-acetylmuramoyl-L-alanine amidase [Bacillaceae]|WP_000135303.1
*peg4292*	33.24	40.36	18	MULTISPECIES: succinate dehydrogenase flavoprotein subunit [Bacillaceae]|WP_000676745.1
*peg1615*	30.53	33.68	18	MULTISPECIES: peptide ABC transporter substrate-binding protein [Bacillaceae]|WP_000823458.1
*peg5937*	28.39	98.60	20	Hypothetical Protein|WP_087969954.1

**Table 6:** Identification of the candidate insecticidal proteins in the KhF strain by LC-MS/MS analysis.

Gene Identity	Protein Molecular Mass (kDa)	Unused ProtScore	Sequence Coverage (%)	Matched Peptides	Hi3 (%) ^2^	Shared Peptides ^3^	Unique Peptides ^4^
*peg5936*	134.32 ^1^	188.93	92.96	171	79.15	23	23
*peg5937*	24.23 ^1^	28.39	98.60	20	4.05	6	2

^1^ The size of the Peg5936 and Peg5937 were obtained from protein sequences derived and predicted by the Glimmer v2 software in the Rast server. ^2^ Hi3 is a quantitative proteomics technique that estimates the quantity of a protein by intensifying the LC-MS data of the tryptic peptides of a protein of interest in the mixture [35,36]. ^3^ Number of peptides with a contribution value higher than 2 that are present in two or more proteins of the described Cry toxins. ^4^ Number of peptides with a contribution value higher than 2 that are present only once in one protein of the described Cry toxins.

## Supplementary Materials

The following are available online at https://www.mdpi.com/2072-6651/12/11/673/s1, Table S1: Predicted virulence factors present in the assembled sequences of the KhF strain using the VF analyzer tool, and Multiple alignment of the candidate insecticidal protein genes and enterotoxin-like genes detected in the genome of the KhF strain, Table S2: Unique peptides for the Peg5936 (KhFB) and Peg5937 (KhFA) proteins detected by LC-MS/MS in the spore/crystal mixture of KhF strain.
